# Influence of Calcium Carbonate Nanoparticles on the Soil Burial Degradation of Polybutyleneadipate-Co-Butylenetherephthalate Films

**DOI:** 10.3390/nano12132275

**Published:** 2022-07-01

**Authors:** Marco Rapisarda, Maria Chiara Mistretta, Michelangelo Scopelliti, Melania Leanza, Francesco Paolo La Mantia, Paola Rizzarelli

**Affiliations:** 1 Istituto per i Polimeri, Compositi e Biomateriali, Consiglio Nazionale Delle Ricerche, Via Paolo Gaifami 18, 95126 Catania, Italy; marco.rapisarda@ipcb.cnr.it (M.R.); melanialeanza.92@gmail.com (M.L.); 2 Dipartimento di Ingegneria, Università di Palermo, Viale delle Scienze, 90128 Palermo, Italy; mc.mistretta@gmail.com; 3 Dipartimento di Fisica e Chimica—Emilio Segrè, Università degli Studi di Palermo, Viale delle Scienze 17, 90128 Palermo, Italy; michelangelo.scopelliti@unipa.it; 4 INSTM, Via Giusti 9, 55100 Firenze, Italy; 5 ATeN Center—Laboratorio Superfici, Film Sottili e Dispositivi, Università degli Studi di Palermo, Viale delle Scienze 18-18/A, 90128 Palermo, Italy

**Keywords:** biodegradable polymers, soil burial test, Ecoflex^®^, poly(butyleneadipate-co-butyleneterephtalate), calcium carbonate, nanoparticles, polymer degradation

## Abstract

A polybutyleneadipate-co-butylenetherephthalate (PBAT) sample, commercially known as Ecoflex^®^, was processed via melt extrusion with CaCO_3_ nanoparticles coated with a hydrophobic coating. Blown films of PBAT and two composites with nanofiller (2% and 5%wt) were prepared and degradation tests in soil at 30 °C up to 180 days were carried out with weight loss measurements. Furthermore, biodegradation test according to ISO 14851 was carried out at 30 °C. The effect of CaCO_3_ on soil burial degradation was assessed by surface wettability and SEM. ATR-FTIR and XPS analyses highlighted chemical modifications induced by soil degradation. CaCO_3_ nanoparticles decreased surface wettability and discouraged the disintegration in soil. Interestingly, SEM images after soil degradation highlighted in the nanocomposite films selective zones of disintegration. XPS showed an increasing peak area C 1s ratio of C–O to C=O with degradation time. Moreover, after the soil burial test, carbonyl index determined by ATR-FTIR increased in both nanocomposites. In fact, the addition of CaCO_3_ leads to a rise in the carbonyl zone due to the presence of the carbonate group. Remarkably, FTIR data after soil degradation showed an enrichment of the aromatic content, a preferential cleavage and erosion of the aliphatic moiety in PBAT films, amplified by the presence of the CaCO_3_ nanofiller.

## 1. Introduction

The use of bio-based and biodegradable plastic materials can provide valuable support to the fruitful transition towards a circular economy. To prevent the accumulation of plastic materials in the open environment, plastic products should be recyclable or biodegradable. In the past decades, research and development activities have afforded commercially available polymers that can fulfill both end-of-life options. Polylactide (PLA), poly(butyleneadipate-co-butylenetherephthalate) (PBAT) and polyhydroxyalkanoates represent the most attractive options in the biodegradable polymer market for their appealing properties. Nevertheless, biodegradable polymers usually have cost and mechanical properties that are not competitive with the traditional plastic materials they aim to replace. These limits can be improved using particulate mineral fillers (e.g., talc, calcium carbonate, mica). CaCO_3_ is one of the most used inorganic reinforcements, which quite efficiently enhances the mechanical characteristics while simultaneously reducing the cost. In fact, the industrial price of CaCO_3_ is lower than that of the most representative biodegradable polymers.

Several studies have been carried out assessing the filling and modification of nanosized CaCO_3_, particularly in biodegradable polyesters and their blends [1,2,3,4,5,6,7]. Effects of fillers on PLA [1,4,6,7], PBAT [2,3,4,7], PHAs [2], polycaprolactone [5], poly(butylene succinate) and copolymers [2] have been investigated.

Films for agricultural applications (e.g., greenhouses or mulching films) are usually made of polyolefins, mainly polyethylene. Nonetheless, the use of biodegradable polymers is increasing. Biodegradable mulching films can be advantageously left on the site, mixed with the soil, and buried in the ground during plowing. The successive biodegradation process gives rise to compost that is a fertilizer for the ground.

Aliphatic polyesters are the most adopted biodegradable materials in agriculture [8]. Numerous studies on biodegradable polymers and blends have been performed to verify their performance, for example in mulching or irrigation pipes [9,10,11,12,13,14]. Nevertheless, both biodegradable plastic items often do not have suitable mechanical performances, and, during their service life, sunlight exposure modifies their properties [15]. Poly(butyleneadipate-co-butylenetherephthalate) (PBAT) is an aliphatic–aromatic biodegradable polyester produced from fossil sources. The global production capacities of PBAT in 2021 represented 19.2% of the bioplastic market and it is set to increase up to 30.0% in 2026 [16]. The approximately 50% of aromatic fraction provides excellent physical properties and the aliphatic fraction supports its degradation in several conditions, including in soil. PBAT is widely used in biodegradable mulching films and extensively studied for many applications [4,17,18,19].

The influence of nanosized CaCO_3_ added, in a percentage ranging from 2 to 20%, in the biodegradable polymer matrix on the biodegradation rate has been investigated. However, studies in the literature have been limited and mainly focused on PLA and its blends [2,4,5,6,7].

Few investigations have been undertaken concerning the biodegradation of PBAT-based CaCO_3_ nanocomposites. In particular, Post et al. studied composites based on a diverse range of biodegradable polymer matrices, including PBAT. They investigated the influence of the inclusion of different fillers on the main mechanical properties of polymer composites. Moreover, they performed a soil disintegration test to identify the effect of both calcium carbonate and talc on biodegradability of poly(3-hydroxybutyrate-co-3-hydroxyhexanoate) (PHBH) and it was monitored by visual observation. Evidence showed that the nature of the filler does affect the biodegradation rate of the matrix polymer. However, no data concerning biodegradability of PBAT nanocomposites have been reported [2]. Rocha et al. used melt extrusion to prepare PBAT/PLA blends reinforced with CaCO_3_ at four different reinforcement compositions (10% and 20%wt). They evaluated the mechanical and thermal properties, crystallinity, water absorption, and soil degradation. The addition of CaCO_3_ improved compatibility between the polymers and the films showed better mechanical properties. Nevertheless, low water absorption and degradation in the simulated soil were detected [4].

Thus, the purpose of this study was to investigate the soil burial degradation of a PBAT sample, commercially known as Ecoflex^®^, processed via melt extrusion with CaCO_3_ nanoparticles. Two nanocomposites with a filler content of 2 (PBAT-2%) and 5%wt (PBAT-5%), selected by previous studies [19], were prepared and degradation test in soil carried out. Weight loss measurements were used to monitor the degradation in soil. Biodegradation test according to ISO 14851 was also carried out. Degradation tests were carried out at 30 °C according to temperature suggestion in international standards [20,21]. The effect of the addition of CaCO_3_ nanoparticles on soil burial degradation was assessed by surface wettability by static contact angle measurements and scanning electron microscopy (SEM) analysis. Attenuated total reflection-Fourier transform infra-red (ATR-FTIR) and X-ray photo electron Spectroscopy (XPS) analyses highlighted chemical modifications induced by soil degradation. In agreement with the literature [17], crystallinity, monitored by differential scanning calorimetry (DSC), increased after degradation in soil.

Overall, the results show that PBAT-CaCO_3_ nanocomposites after biodegradation in soil show a higher content of calcium carbonate and aromatic fraction than in the initial polymer sample.

## 2. Materials and Methods

### 2.1. Materials and Preparation of the Nanocomposite Films

The polymer matrix used in this work was a polybutylene-co-adipate-therephthalate, (PBAT) commercially known as Ecoflex^®^ C1200 (BASF—Ludwigshafen am Rhein Germany). The PBAT sample has a melt flow index of 2.7–4.9 g/10 min (2.16 kg at 190 °C) and density 1.25–1.27 g cm^−3^ according to the manufacturer’s specifications [22].

The filler was a nanosized calcium carbonate commercially known as Socal 312 (Solvay S. A.—Neder-Over-Heembeek, Brussels, Belgium). It is an ultrafine, white, and odorless, coated precipitated calcium carbonate with 99.6% CaCO_3_ content, main particle diameter of 70 nm and specific surface area 17 m^2^/g. The nanoparticles are coated with a hydrophobic coating [23] made of a mixture of calcium stearate (CaSt) and calcium palmitate (CaPa) as reported in the literature [19].

The pellets of the polymer were dried at 70 °C under vacuum for at least 4 h before use, while calcium carbonate was dried for 12 h at 90 °C. PBAT and the two composites with a filler content of 2% (PBAT-2%) and 5% (PBAT-5%) were processed in a co-rotating intermeshing twin-screw extruder (OMC, Italy), having a diameter D = 19 mm and a length-to-diameter ratio L/D = 35, using a shear stress screw profile. The temperature profile for all the samples was 150–160–160–170–170–180–180 °C (at the die), the screw speed set at 230 rpm and the speed of the gravimetric feeder set at 12 rpm.

All the materials obtained after this processing step were pelletized and fed to a single screw extruder (Brabender—Duisburg, Germany) with a diameter D = 19 mm, L/D = 25 and using a thermal profile 150–160–160–180 at a speed of 75 rpm. The extruder was equipped with a film-blowing unit (Brabender, Germany) that allowed preparation of blown films with an average thickness of about 40 μm.

The commercial soil (Geolia, Virgoplant—Piacenza, Italy) was composed of peat, composted and non-composted plant amendments (pH in H_2_O = 6.5; electric conductibility 0.4 dS/m).

### 2.2. Film Degradation Studies

#### 2.2.1. Soil Burial Test

Tests were carried out at 30.0 ± 0.1 °C, under moisture-controlled conditions. Triplicate specimens of film samples were placed in darkened vessels containing a multi-layer substrate [24,25]. Filter paper sample was used, as a positive control. The film samples were cut into 2 cm × 2 cm pieces. Specimens of all films (initial weight 5–10 mg, filter paper ≅ 25 mg; Mettler Toledo MX5, readability/d = 1 µg) were sandwiched between two layers of a mixture of milled perlite (50 g) and commercial soil (200 g), moistened with 100 mL of distilled water. The bottom and top layers were filled with 60 g of perlite moistened with 120 mL of distilled water. Perlite was used for increasing aeration to the soil and the amount of water retained. A flow of moistened air was supplied from the bottom of each vessel every 24 h for 15 min. After regular intervals all the film samples were removed, cleaned, brushed softly, washed with distilled water several times, dried under vacuum in the presence of P_2_O_5_ at room temperature, to constant weight and then photographed. The degree of degradation was evaluated by weight loss (WL) by using the following equation:

WL (%) = (Wi − Wt)/Wi × 100,(1)

where Wi is the initial weight of the sample and Wt is the weight after the established time.

Contact angle measurements, SEM, DSC, XPS and ATR-FTIR analyses at least in triplicate were carried out on the recovered films after soil burial test.

#### 2.2.2. Biodegradation Degree

Total organic carbon (TOC) is a necessary parameter for carrying out tests to determine the degree of biodegradation of a sample. It is preliminarily determined to establish the amount of sample to be used in the biodegradation test. The value of total organic carbon was carried out by using a TOC-L CSH (Shimadzu Corporation—Kyoto, Japan) equipped with a solid sample module SSM-5000A. Two aliquots of the same sample were weighed and placed in ceramic boats inside the two furnaces of the TOC analyzer, the first was kept at 900 °C for the determination of total carbon and the other, in which the sample is first acidified with phosphoric acid for the determination of inorganic carbon, at 200 °C. The carbon dioxide produced is determined by an IR detector. The determination of the total organic carbon is carried out by subtracting the fraction of the inorganic carbon from the value of the total carbon.

Degree of aerobic biodegradation was determined by respirometric method according to ISO 14851:1999 [26]. Biochemical oxygen demand (BOD) in a closed respirometer was measured with an OxiTop^®^ system. A sample of paper was used as a positive reference. All the samples were introduced in amber bottles, together with 164 mL of test medium, under magnetic stirring and placed in an incubator, at a constant temperature (30 ± 1 °C) for about 3 months [27]. The medium consisted of salts dissolved in water ([KH_2_PO_4_] = 85.0 mg/L; [K_2_HPO_4_] = 217.5 mg/L; [Na_2_HPO_4_ ● 2 H_2_O] = 334.0 mg/L; [NH_4_Cl] = 5.0 mg/L; [MgSO_4_ ● 7H_2_O] = 22.5 mg/L; [CaCl_2_ ● 2 H_2_O] = 36.4 mg/L; [FeCl_3_ ● 6H_2_O] = 0.25 mg/L) and an appropriate volume of inoculum. The salts guarantee the right amount of nutrient for micro-organisms and for maintaining the pH at a value of 7.4; the inoculum was obtained by filtering the supernatant of a suspension of 10 g of mature compost in 100 mL of test medium. The degree of biodegradation was calculated according to the Equation:

Biodegradation (%) = (BODs/ThOD) × 100,(2)

where ThOD is the theoretical oxygen demand, in mg/g of test material; BODs is the specific BOD, in mg/g of test material:

BODs = (BODt − BODb)/ρt × 100,(3)

and BODt is the BOD of the flask containing the test material, in mg/L, whereas BODb is BOD of the blank flask, in mg/L and ρt is the concentration of the test material in the flask, in mg/L [26].

### 2.3. Characterization

#### 2.3.1. Static Contact Angle (SCA)

Using a contact angle goniometer (OCA15EC, Dataphysics—Filderstadt, Germany), the surface wettability values of the samples at room temperature were measured. Static contact angle (SCA) values were determined by dropping 2 µL of water from a micro syringe onto the film surfaces and analyzing by software (SCA 20) the images taken by the connected video camera. The samples were previously equilibrated for 30 min to eliminate possible interference and then the SCA was measured. The films were kept flat using a sample holder that allowed their correct positioning and stretching. At least five measurements were carried out for each sample to ensure repeatability of the experiments.

#### 2.3.2. Scanning Electron Microscopy (SEM)

The samples, previously metalized, were characterized by scanning electric microscopy Thermo Phenom Prox (Thermo Fisher Scientific—Waltham, MA, USA) desktop scanning electron microscopy (SEM) combined with a fully integrated energy-dispersive X-ray detector (Silicon Drift Detector) to evaluate the morphology.

#### 2.3.3. Differential Scanning Calorimetry (DSC)

Differential scanning calorimetry was performed using ≅ 3 mg samples under nitrogen flow with a TA Instrument Q100. For all samples analyzed, the temperature programming was as follows: heat from 25 °C to 200 °C at 10 °C/min, cool down to −20 °C at 50 °C/min and heat to 200 °C at 10 °C/min. The melting temperatures (Tm) were taken as the peak temperature of the melting endotherm. The crystalline fraction Xc (%) was calculated by the equation:

Xc = (ΔHm/ΔHm_100_) × 100, (4)

where ΔHm is the enthalpy of fusion of the sample/composites. The theoretical enthalpy of 100% (ΔHm_100_, PBAT = 114 J/g) has been taken from the literature [28].

#### 2.3.4. X-ray Photoelectron Spectroscopy (XPS)

XPS spectra were collected for all the samples (virgin, after 120 and 180 days of soil burial degradation) for the different CaCO_3_ content (0, 2 and 5%). The spectra were acquired in the C 1s region by means of a PHI5000 VersaProbe II scanning microprobe (ULVAC-PHI, Inc., Chigasaki, Japan), working with an aluminum anode (K_α_ = 1486.6 eV) and using a ⌀ 100 μm, 25 W, 15kV beam. The surfaces of the samples were kept at a 45° with respect to the analyzer, operating in fixed analyzer transmission (FAT) mode. Due to the high thermal sensitivity of PBAT—at least, in the XPS acquisition conditions—special care was given to find optimal conditions to avoid thermal degradation and/or wearing of the surfaces, as evidenced by secondary X-electrons imaging (SXI), used to check the integrity of the sample before and after the XPS spectral acquisition. Thus, collected spectra were acquired with an energy resolution of 0.1 eV, averaging over a 5 min max illumination (8 sweeps per sample) to avoid overheating. Spectra were collected at the ATeN Center facility, University of Palermo.

#### 2.3.5. Attenuated Total Reflection-Fourier Transform Infra-Red (ATR-FTIR)

ATR spectra were collected using an FT-IR spectrometer (Perkin Elmer, Norwalk, CT, USA) equipped with attenuated total reflection (ATR). The spectra were recorded in a wavenumber range of 4000 cm^−1^ to 400 cm^−1^, averaged over 8 scans. Since it is difficult for PBAT to identify a reference peak to evaluate the changes related to carbonyl groups, due to the overlapping of carboxylic and aromatic stretching signals, a ratio (carbonyl index) between the area relative to the carbonyl zone and the area relative to the stretching of methyl groups and methylenes was considered. So, the areas between 1550 cm^−1^ and 1850 cm^−1^ and the area between 2750 cm^−1^ and 3050 cm^−1^ were calculated [29,30]. In the same way, to evaluate the changes related to aromatic groups, a ratio (aromatic index) between the area relative to the aromatic groups (680 cm^−1^ and 780 cm^−1^) and the area relative to the aliphatic stretching was considered.

## 3. Results and Discussion

### 3.1. Degradation Tests

The degradation of plastic materials, traditional and biodegradable, involves different processes promoted by one or more environmental factors (i.e., heat, light, microorganisms) or chemicals. Biodegradation is a three-step process: in the first stage, macromolecular chains are depolymerized into monomers and oligomers; in the second, the monomers and oligomers are taken up as biomass; and in the last step, the respiration of biomass consumes O_2_ and produces CO_2_ and H_2_O (under aerobic conditions). All the standardized methods for determining biodegradation are addressed on the measurement of the conversion into carbon dioxide of the organic carbon initially present in the plastic by the oxygen. On the other hand, in the literature most of the papers concerning biodegradation of polymers and composites are based on weight loss [31,32,33] which is accepted as a measurement of biodegradability of plastic films.

In this paper, we performed both a soil burial test by using WL as a biodegradability index and a respirometric test according to an international standard method. The soil burial degradation test was carried out by sandwiching the polymer films between layers of a mixture of milled perlite and commercial soil to simulate soil degradation after their use lifetime. Perlite was used to increase the amount of water retained and accelerate degradation in soil [24,25]. WL was used as a parameter for evaluate biodegradability [31,32,33].

**Figure 1:**
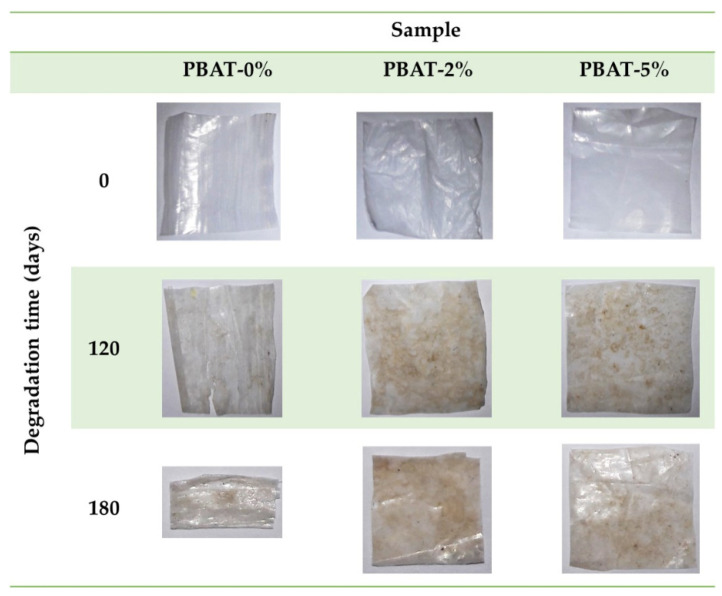
Representative photos of PBAT and nanocomposite film samples, before and after soil burial degradation test.

Figure 1 shows some representative photographs of the film samples recovered after 0, 120 and 180 days of the burial test. All the nanocomposite film portions appear almost intact, while the degradation of PBAT-0% samples produced embrittlement, and some longitudinal cracks after 120 days.

**Figure 2:**
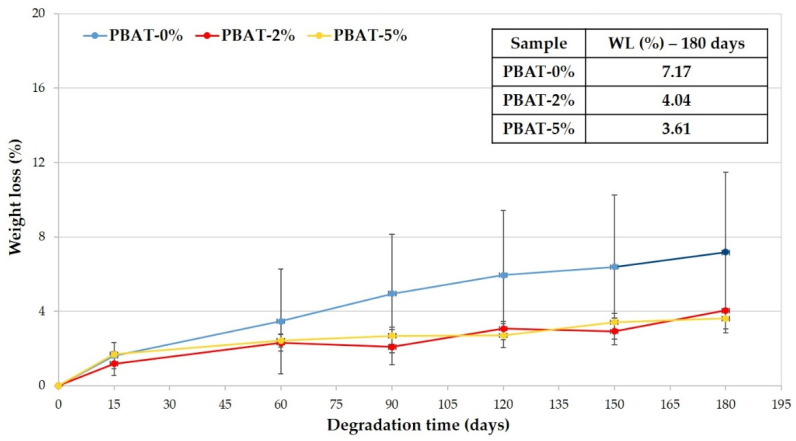
Weight loss percentage vs. degradation time of PBAT polymer samples.

In Figure 2 the average weight losses of the PBAT samples is reported vs. the soil burial time. The WL increases and kinetic of biodegradation decreases with burial degradation time slower than for other samples (i.e., Mater-Bi^®^ by Novamont, Bio-Flex^®^ by FKUR, Ecovio^®^ by BASF) that were previously tested in similar conditions [12,13]. Data highlight that the higher the CaCO_3_ percentage is the lower is the WL reached in 180 days (Figure 2).

**Figure 3:**
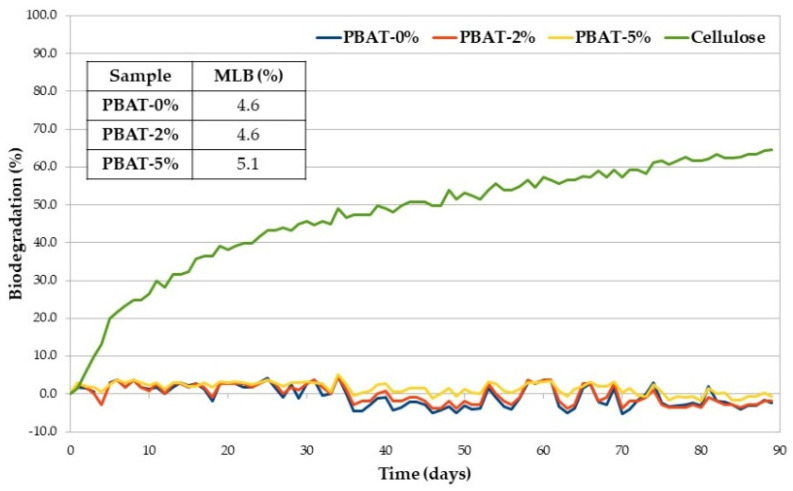
Average biodegradation (%) as a function of time of PBAT samples and cellulose (positive reference).

A low susceptibility to soil micro-organisms was confirmed in the respirometric test carried out according to ISO 14851 [26] and based on BOD measurement [27]. Figure 3 shows the average percentage of biodegradation (at 30 °C) plotted as a function of time of positive reference (cellulose), PBAT-0%, PBAT-2% and PBAT-5%, together with the maximum level of biodegradation (MLB) detected within 90 days. The selected test temperature is not the preferred temperature in ISO 14851 but was selected to be comparable with the soil burial degradation temperature. Biodegradation traces are typically characterized by a lag phase, which is the interval from the start of the test until a clear biodegradation (i.e., 10%) can be recorded; a biodegradation phase, in which the maximum degradation takes place; and a plateau phase, in which biodegradation is almost completed. Degree of biodegradation of cellulose at the end of the test went over the limit value of 60%, required by the ISO 14851 to prove the validity of the test [26]. All the PBAT samples, with and without CaCO_3_, showed a very low oxygen consumption over time due to the action of bacteria on the organic carbon present in the matrices and determined by TOC analysis. The maximum level of biodegradation of PBAT-5% is slightly higher than those of the other two samples. Whenever WL is used to monitor the biodegradation of polymer samples, just the first step of the process is involved, i.e., macromolecular chain depolymerization into monomers and oligomers that are eroded from the surface [33]. The generated low molecular weight products should cross the cellular membrane and then be used by the microbial cells in their metabolic process, consuming O_2_ (monitored in our respirometric test) and producing CO_2_ and H_2_O. The last two steps and their kinetics depend on the structure of the monomers and oligomers formed in the first step that can be eroded from the film surface but not necessarily metabolized with the same rate they were generated.

### 3.2. Surface Wettability

**Figure 4:**
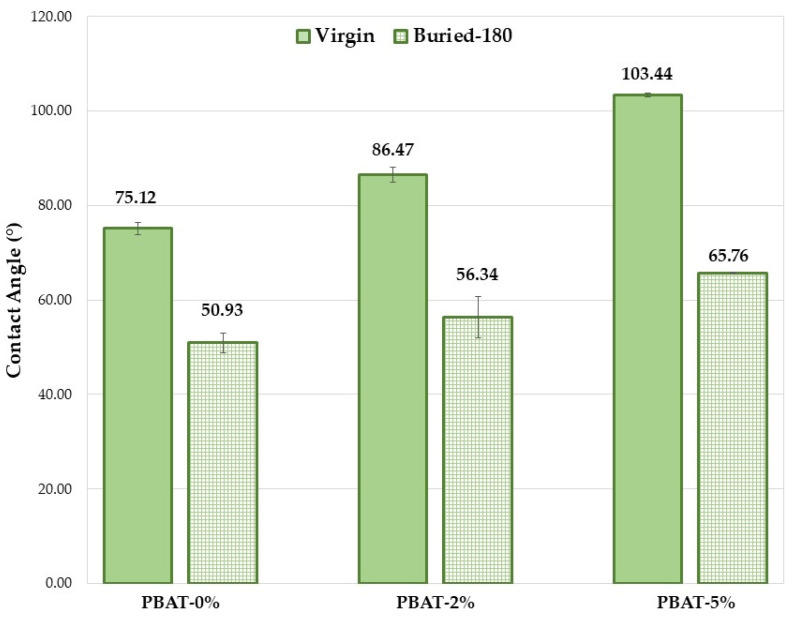
Average static contact angle values for the PBAT film samples (180 days).

The hydrophilicity/hydrophobicity of a composite surface can obviously have an important influence on the hydrolysis rate. A higher hydrophilicity provides a stronger water adsorption capacity on the material surface that leads to a greater hydrolysis rate. The wettability of the PBAT film surfaces before and after soil burial test was determined by static contact angle (SCA) measurements. The SCA values clearly increase with the content of CaCO_3_ nanoparticles coated with a hydrophobic coating of CaSt and CaPa (Figure 4). Chemical modifications on the surface of the thin films (COOH and OH end groups) induced by soil degradation increase plastic samples wettability, highlighted by SCA decrease (Figure 4) and, consequently, the microbial susceptibility is encouraged, as observed in other investigations [12,13]. However, the increase in surface wettability induced by soil burial test is more pronounced for the PBAT-5% (Figure 4, ∆CA ≅ 40°). SCA decrease is related to the formation of hydrophilic chain ends. Oligomeric species produced by hydrolysis are progressively removed by surface erosion. Hypothetically, CaCO_3_ could keep these and partially delay their removal from the surface.

### 3.3. Surface Morphology

**Figure 5:**
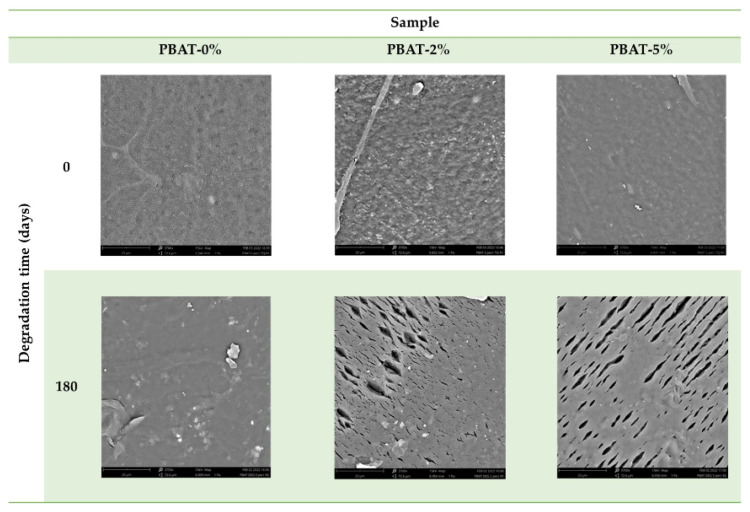
SEM images of PBAT film samples without and with addition of CaCO_3_, before and after soil burial degradation (180 days).

The surface morphology of PBAT film nanocomposites before and after degradation were observed by SEM (Figure 5). The surface of PBAT-0% is smooth before and after degradation. After degradation, several cracks, with a preferential direction, and cavities as well as eroded regions are evident in the surface of PBAT-2% and PBAT-5%. This is most likely due to a regular surface erosion mechanism in PBAT-0%, while in PBAT nanocomposites, the CaCO_3_ nanoparticles, being not uniformly distributed, could induce a preferential erosion in some zones and the formation of parallel cracks (5–20 µm, Figure 5).

### 3.4. Thermal Analysis

Table 1 reports the Tm and Xc of PBAT nanocomposite films before and after soil burial degradation. The addition of CaCO_3_ nanoparticles reduces both the Tm and Xc. This trend agrees with data in the literature concerning PLA nanocomposites and its blends with PBAT where, however, a higher percentage of CaCO_3_ nanoparticles was used [4,6].

**Figure 6:**
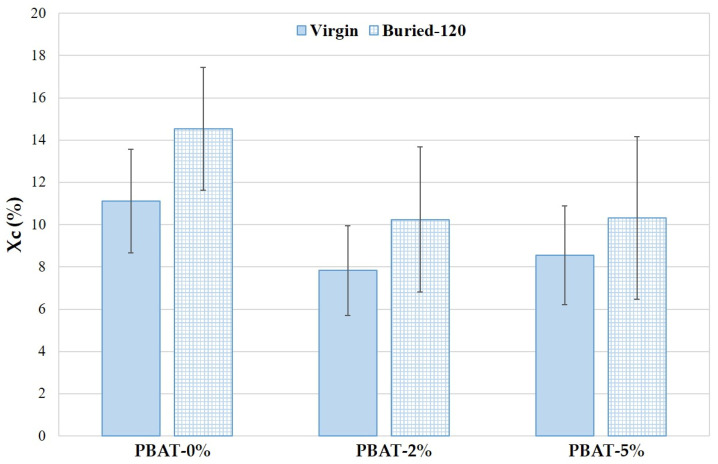
Crystallinity (Xc) of PBAT and nanocomposites, before and after degradation (120 days).

The Xc trend of PBAT nanocomposites with different CaCO_3_ nanoparticle percentage, before and after degradation, is reported in Figure 6. The crystallinity of PBAT nanocomposites is lower than PBAT-0%. The CaCO_3_ nanoparticles can be beneficial in promoting the formation and growth of crystal nuclei as well as the crystalline speed due to their nucleation agent effect, as reported in studies on PLA [1]; however, the movement of the molecular chains can be limited, and the crystallization reduced. As a result, the crystalline degree in PBAT nanocomposites decreases slightly with the presence of CaCO_3_ nanoparticles. The crystallinity increases on film residues after 120 days of soil burial degradation (Figure 6). The reduction of the amorphous fraction is more pronounced for PBAT-0%. These data agree with the literature [18]. The biodegradation of amorphous regions by micro-organisms and enzymes are faster than the crystalline ones.

### 3.5. Structural Modifications Induced by Soil Burial Degradation

The chemical modifications induced by soil burial degradation of the surfaces of PBAT nanocomposite films were investigated by XPS and ATR-FTIR.

#### 3.5.1. XPS Results

Owing to the nature of the samples and the degradation procedure, an accurate XPS analysis concerning all the elements observed in a survey scan would result in false positives, especially in the case of an oxygen analysis—there are too many sources for superficial hydration. For such a reason, our analysis concentrated in the C 1s region for all studied samples. Figure S1 shows the C 1s core-level spectra of PBAT, PBAT-2% and PBAT-5% before and after 120 and 180 days of soil burial degradation together with the PBAT repetitive unit. As expected, for all samples a pattern of three main contributors may be observed: C–C/C–H (Binding Energy, BE = 284.8 eV, used as an energy reference); C–O–C (BE expected: ≅ 286 eV; BE found: 286.2–286.4 eV); O–C=O (BE expected: ≅ 288.5 eV; BE found 288.3–288.7 eV). The peak labeled as C–O–C is, in fact, the sum of all species containing a single C−O bond, thus both ester alkoxy carbon and its hydrolyzed counterpart, the alcoholic moiety; whereas the peak labeled as C=O indicates the carboxylic carbon, thus both ester and degraded (hydrolyzed) form. Due to the relatively low abundance, the added carbonate from CaCO_3_ does not alter the carboxylate signal (in the case of the higher carbonate concentration, the contribute area would amount to less than 5% of the carboxylate peak); as such, the carbonate contribution was neglected in the data analysis.

**Figure 7:**
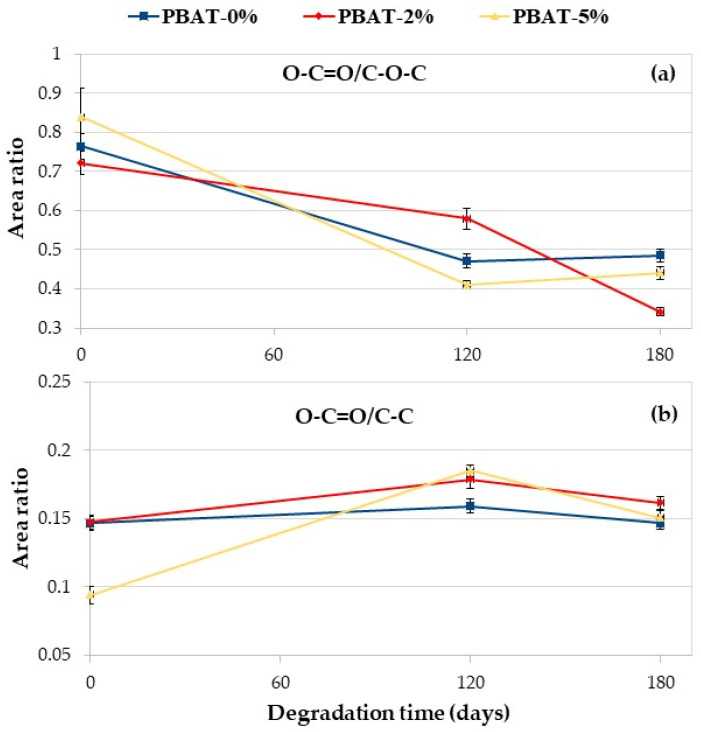
XPS C 1s area ratio vs. degradation time: (**a**) O–C=O/C–O–C; (**b**) O–C=O/C–C.

The degradation process, because of the cleavage of the ester linkages, produces hydroxyl- and carboxyl-terminated PBAT chain ends. As previously indicated, due to the superficial nature of the XPS technique, a direct evaluation of the abundance of differently oxidized carbons could be misleading due to adsorbed species. For such a reason, we decided to minimize the adventitious contributions evaluating the ratio between the areas of the peaks (i.e., the relative abundance). Areas were evaluated by means of conventional data fitting, by means of the software MultiPak 9.9.2 (ULVAC-PHI) using a Shirley background and symmetric Gauss–Lorentz lineshapes for the peaks; fit standard deviations were evaluated by means of the numpy python package [34]. The ratio of peak areas O–C=O/C–O–C and O–C=O/C–C of the PBAT nanocomposites vs. degradation time in soil is reported in Figure 7. Data further confirm the degradation of polymer films. The area ratio O–C=O/C–O–C decreases with the degradation time for all the samples (Figure 7a), in agreement with the literature [18]. The area ratio of O–C=O/C–C is almost constant for the PBAT-0% while it increases for the nanocomposites after 120 days of degradation in soil markedly for PBAT-5% (Figure 7b). Oligomers are eroded from the surface and the CaCO_3_, being embedded in the polymer matrix, increases the O–C=O on the film surface. After 180 days, the area ratio of O–C=O/C–C slightly decreases but is higher than the initial value (Figure 7b).

#### 3.5.2. ATR-FTIR Results

Figure S2 shows the ATR-FTIR spectra of virgin PBAT and the two nanocomposites in the full wavenumber range (Figure S2a), 1550–1900 cm^−1^ range (Figure S2b) and 600–800 cm^−1^ range (Figure S2c). As observed in the literature, the peaks present in the area between 1600 cm^−1^ and 1850 cm^−1^ correspond to the carbonyl bond C=O, the peak present in the area between 600 and 800 cm^−1^ correspond to the aromatic C–H, while the peaks in the area between 2750 cm^−1^ and 3050 cm^−1^ are due to the symmetric and asymmetric stretchings, ν_s_ and ν_a_, of CH_2_ and CH_3_ [19]. The addition of calcium carbonate leads to an increase in the carbonyl zone due to the presence of the carbonate group (Figure S2b).

**Figure 8:**
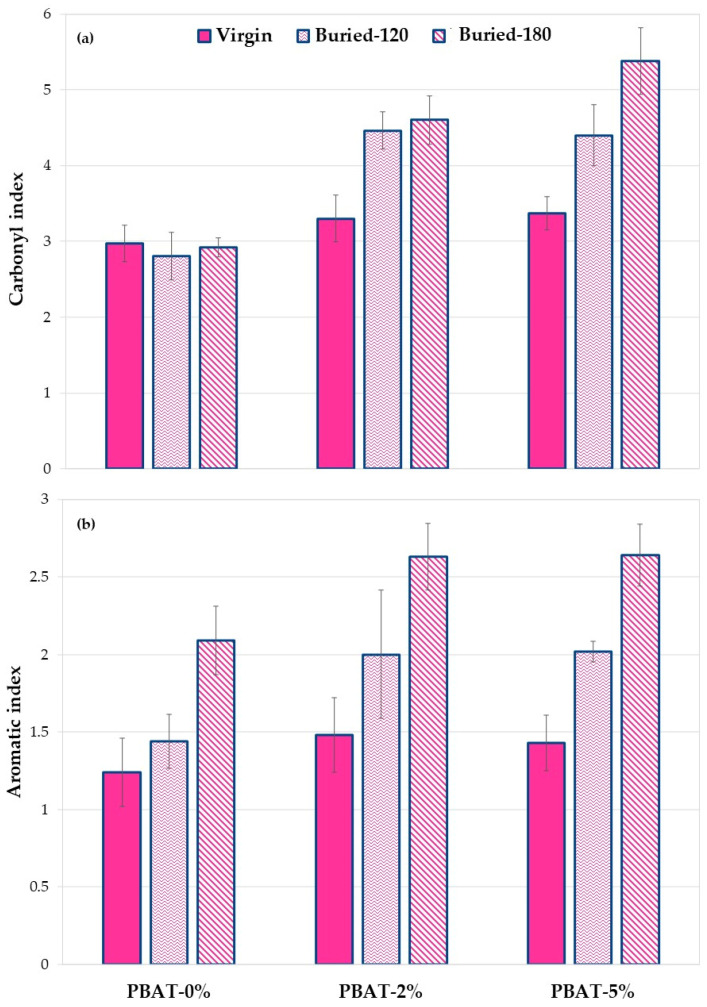
(**a**) Carbonyl and (**b**) aromatic index values for PBAT and nanocomposites film samples, before and after soil burial degradation (120 and 180 days).

As mentioned above, since it is difficult to identify a reference peak to evaluate the changes related to functional groups, ratios between the areas relative to the carbonyl or the aromatic zones and the area relative to the stretching of methyl- and methylene-groups were considered, as these have been frequently used previously [12,13,29,30]. The ratios between the above areas (carbonyl index and aromatic index) for virgin PBAT and nanocomposites, before and after the soil burial test, were then calculated. The data obtained are shown in Figure 8.

The results in Figure 8a show, as mentioned above, that the addition of calcium carbonate results in an increase in the area value at 1600–1850 cm^−1^ due to the C=O groups present in the carbonate. This result is evident both with the addition of 2% and with the addition of 5%.

Following the burial of the PBAT-0%, a slight decrease in the area ratio is observed. This is due to biodegradation phenomena that involve a modification of the ester group following the phenomena of hydrolytic scission. A different behavior is observed in the two nanocomposites with calcium carbonate. In fact, when adding 2% of calcium carbonate and after 120 days of soil burial degradation, an increase in the ratio between the areas is observed. This increase could suggest a decrease in degradation kinetics thus showing a protective effect of calcium carbonate. Calcium carbonate nanoparticles, in fact, probably because of their hydrophobic coating, make the polymer surface less susceptible to bacterial degradation. This effect is more pronounced in the composite with 5% of calcium carbonate. The data clearly show that the PBAT nanocomposite samples after biodegradation in soil have a higher calcium carbonate content, reproducing the same result highlighted by XPS analysis (Figure 7).

Figure 8b shows the aromatic indexes of the PBAT sample before and after soil burial degradation test at 120 and 180 days. Noteworthily, data clearly highlight a gradual enrichment of the aromatic content on the surface of the recovered films. This increase is more pronounced for the nanocomposite samples, particularly after 120 days of degradation. This result is a first proof of a preferential cleavage and erosion of the aliphatic moiety in PBAT film samples that could be amplified by the presence of the CaCO_3_ nanofiller. These preliminary results show a trend of the ratio of selected functional groups. Further research based on more accurate quantitative analysis will be carried out.

## 4. Conclusions

Degradation in soil of blown films of a commercial PBAT (PBAT-0%) and two nanocomposites with a calcium carbonate (CaCO_3_) content of 2% (PBAT-2%) and 5% (PBAT-5%) was studied by weight loss and a respirometric test. Soil burial degradation test at 30 °C was carried out sandwiching the polymer films between layers of a mixture of milled perlite and commercial soil to simulate degradation in soil after the use lifetime. CaCO_3_ nanoparticles reduced the disintegration in soil of the nanocomposite film samples. In fact, WL reached in 180 days decreased with the increase of CaCO_3_ content. All the PBAT samples showed a very low O_2_ consumption along the time.

Static contact angle, SEM, DSC, XPS, and ATR-FTIR analyses were carried out to evaluate the effect of the addition of CaCO_3_ nanoparticles on soil burial degradation of the PBAT films. The SCA values clearly increased with the content of CaCO_3_ nanoparticles covered with a hydrophobic coating of CaSt and CaPa. Chemical modifications on the surface of the thin films induced by soil degradation (i.e., hydroxyl and carboxylic acid end groups) increased samples wettability, highlighted by SCA decrease, thus encouraging the microbial susceptibility. Remarkably, SEM images after soil degradation showed selective zones of degradation in the PBAT-2% and PBAT-5% films. After 180 days of soil burial degradation, several parallel cracks are evident in the surface of PBAT-2% and PBAT-5%. This was attributed to a uniform surface erosion mechanism in PBAT-0%, while in PBAT nanocomposites, the CaCO_3_ nanoparticles, not homogeneously distributed, induce a preferential erosion in some areas. The Tm and crystallinity of PBAT nanocomposites were lower than PBAT-0%. Crystallinity increased on film residues after soil burial degradation. This agrees with the literature since the biodegradation of amorphous regions by micro-organisms and enzymes is faster than the crystalline ones. Both ATR-FTIR and XPS analyses highlighted chemical modifications induced by soil degradation. In agreement with the literature, XPS showed an increasing peak area C 1s ratio of C–O to C=O with degradation time. Moreover, after the soil burial test, there was no difference of carbonyl index determined by ATR-FTIR for virgin PBAT, while in both nanocomposites it increased. In fact, the addition of CaCO_3_ nanoparticles leads to a rise in the carbonyl zone due to the presence of the carbonate group. The results related to the aromatic CH show an increase in aromatic/aliphatic ratio at the surface, especially for the nanocomposites. This result represents a first proof of a preferential erosion of the aliphatic moiety in PBAT film samples that could be amplified by the presence of the CaCO_3_ nanofiller.

Overall, films have a higher carbonate and aromatic content than the initial samples after biodegradation in soil nanocomposite. Moreover, the results reveal that CaCO_3_ reduced the disintegration rate in polymer films. The effects shown in this work could be relevant in the commercial field since CaCO_3_ is commonly used in biodegradable and compostable carrier bags and other commercial items.

## Figures and Tables

**Table 1:** Melting temperatures (Tm) and crystallinity (Xc) of PBAT and nanocomposite films, before and after soil burial degradation.

	Tm (°C)		Xc	
Sample	t_0_	t_120_	t_0_	t_120_
PBAT-0%	127.74 ± 1.15	127.42 ± 1.59	11.11 ± 2.45	14.54 ± 2.91
PBAT-2%	123.95 ± 0.57	123.29 ± 2.08	7.83 ± 2.13	10.24 ± 3.44
PBAT-5%	121.32 ± 1.04	121.86 ± 2.36	8.55 ± 2.34	10.32 ± 3.85

t_0_ = virgin film samples; t_120_ = buried film samples (120 days).

## Data Availability

The data presented in this study are available on request from the corresponding authors.

## Supplementary Materials

The following supporting information can be downloaded at: https://www.mdpi.com/article/10.3390/nano12132275/s1, Figure S1: XPS C 1s spectra of PBAT samples (**a**–**c**) before and (**d**–**i**) after soil burial degradation. Figure S2: FTIR-ATR spectra of PBAT and nanocomposites (**a**) in the whole wavelength range, (**b**) 1550–1900 cm^−1^ range and (**c**) 600–800 cm^−1^ range.
