# Supplementary material for: Influence of Calcium Carbonate Nanoparticles on the Soil Burial Degradation of Polybutyleneadipate-Co-Butylenetherephthalate Films
Source: Nanomaterials (Basel). 2022 Jul 1;12(13):2275. doi: 10.3390/nano12132275 (PMC9268366; doi:10.3390/nano12132275)
Supplement: Supplementary file 1 [file nanomaterials-12-02275-s001.zip › nanomaterials-1776226-supplementary.pdf]

Supplementary materials

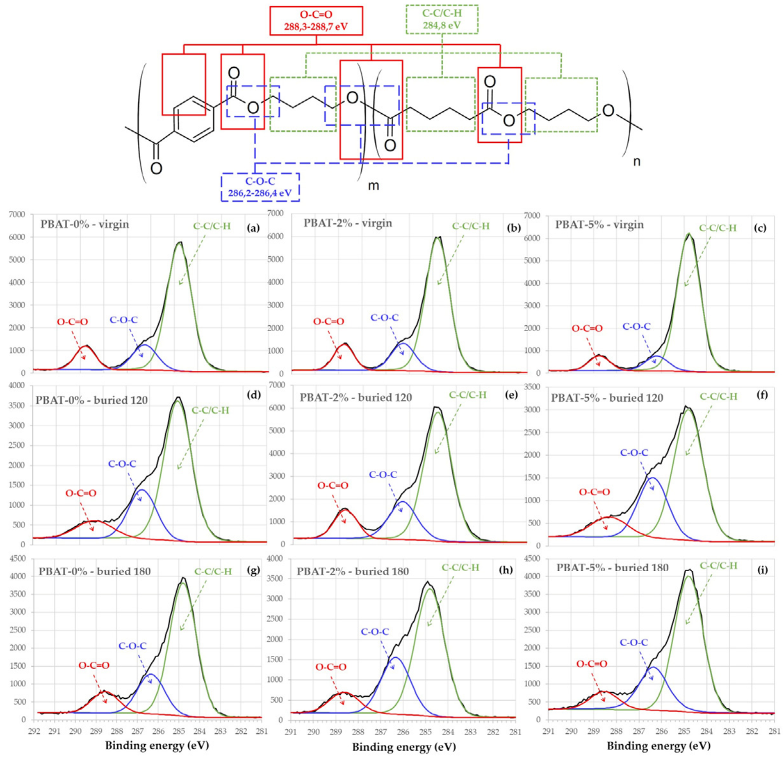

Figure S1. XPS C 1s spectra of PBAT samples (**a**–**c**) before and (**d**–**i**) after soil burial degradation.

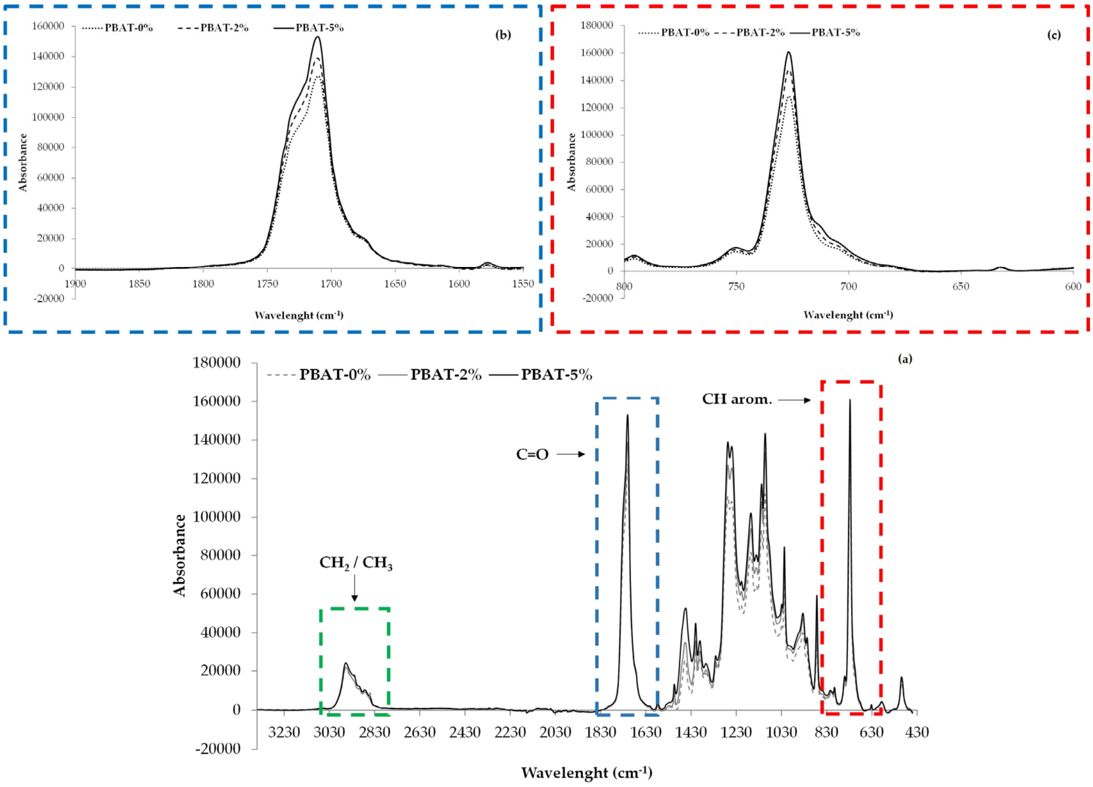

Figure S2. FTIR-ATR spectra of PBAT and nanocomposites (**a**) in the whole wavelength range, (**b**) 1550–1900 cm^{-1} range and (**c**) 600–800 cm^{-1} range.
